# Efficacy and safety of Braun enteroenterostomy in patients undergoing pancreatoduodenectomy: a systematic review and meta-analysis

**DOI:** 10.1007/s00464-026-13370-8

**Published:** 2026-09-23

**Authors:** Marios Alogakos, Ahmed Ghani, Stefan Bitar, Georgia Angeliniades, Konstantinos Manousidis, May Y. Hajeir, Lamees M. Al Darwashi, Christian A. Than, Hayato Nakanishi, Alexandra M. Roch, Eugene P. Ceppa

**Affiliations:** 1 Department of Surgery, Division of Surgical Oncology, Indiana University School of Medicine, 545 Barnhill Dr, EH541, Indianapolis, IN 46202 USA; 2 School of Medicine, University College Dublin, Dublin, D04 C1P1 Ireland; 3 School of Medicine, University of Nicosia, 2417 Nicosia, Cyprus; 4 Cambridge University Hospitals NHS Foundation Trust, Cambridge, CB2 OQQ England; 5 School of Biomedical Sciences, The University of Queensland, St Lucia, 4072 Australia; 6 Department of Surgery, Mayo Clinic, Rochester, MN 55905 USA

**Keywords:** Braun enteroenterostomy, Pancreatoduodenectomy, Delayed gastric emptying, Postoperative pancreatic fistula, Meta-analysis

## Abstract

**Background:**

Delayed gastric emptying (DGE) is a common complication after pancreatoduodenectomy (PD). Braun enteroenterostomy (BEE) has been proposed as an adjunct to reduce DGE. This meta-analysis aims to evaluate the safety and efficacy of BEE in patients undergoing PD.

**Methods:**

CINAHL, PubMed, Embase, Scopus, Web of Science, and Cochrane were searched from inception until November 2025. The pooled mean and proportions were analyzed using a random-effects model. This review was prospectively registered with PROSPERO (CRD420251160291).

**Results:**

Seventeen studies comprising 2731 patients were included, consisting of 1407 (51.5%) patients with BEE and 1324 (48.5%) without BEE. Serious morbidity was similar between groups (OR = 0.66, 95% CI 0.36, 1.19, *I*^2^ = 34%). The rate of reoperation was lower in patients with BEE (OR = 0.35, 95% CI 0.20, 0.64, *I*^2^ = 0%). The rates of overall and clinically relevant DGE (Grades B+C) were lower in patients with BEE (OR = 0.39, 95% CI 0.28, 0.54, *I*^2^ = 33% and OR = 0.36, 95% CI 0.23, 0.57, *I*^2^ = 45%). Similarly, the rates of overall and clinically relevant (Grades B+C) postoperative pancreatic fistula (POPF) were lower in patients with BEE (OR = 0.68, 95% CI 0.49, 0.94, *I*^2^ = 42% and OR = 0.60, 95% CI 0.41, 0.88, *I*^2^ = 34%). Operative time was higher in patients with BEE (MD = 12.6 min; 95% CI 0.9, 24.3, *I*^2^ = 58%), while hospital stay was shorter (MD = − 3.4 days, 95% CI − 5.6, − 1.1, *I*^2^ = 75%). On subgroup analysis including four randomized controlled trials (RCT) (262 patients), no significant difference was observed between groups for any of the above outcomes.

**Conclusions:**

BEE appears to be a safe and effective adjunct in selected adult patients undergoing PD. Despite a higher operative time, BEE was associated with a decreased rate of DGE and POPF. However, these findings were not confirmed in a subgroup analysis of four RCTs and should therefore be interpreted with caution. Further randomized prospective studies are required to assess the causative effect of BEE and confirm these promising findings.

**Graphical abstract:**

**Figure:**
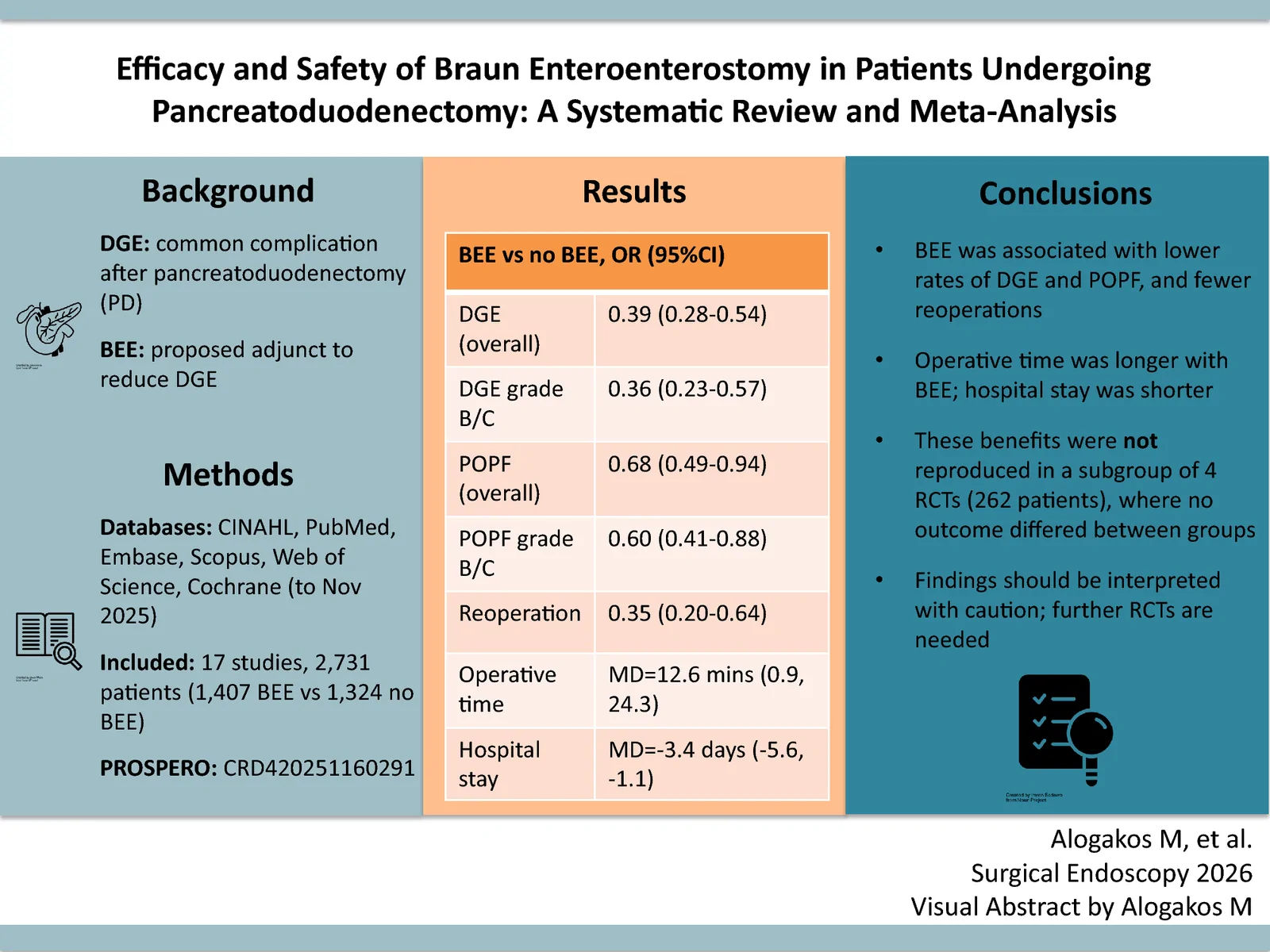

**Supplementary Information:**

The online version contains supplementary material available at https://doi.org/10.1007/s00464-026-13370-8.

Pancreatoduodenectomy (PD) remains the standard surgical treatment for various diseases of the pancreatic head and periampullary region. Important refinements in perioperative care and operative techniques have been implemented in the modern era, resulting in substantially lower in-hospital mortality rates of < 4% [1, 2]. Despite these improvements, postoperative morbidity remains substantial in PD, with rates ranging from 30 to 60% [2–4]. Delayed gastric emptying (DGE) is among the most frequent complications of PD. It has been reported to affect as high as 62% of patients, although the incidence notably varies widely across the literature [5–9].

The pathophysiology of DGE following PD is incompletely understood and is likely attributed to several interacting physiological disturbances. Although rarely fatal, DGE can markedly prolong hospitalization, increase healthcare costs, and negatively impact patient quality of life and psychosocial wellbeing [10]. Given the troublesome nature of DGE, adjunctive interventions like Braun enteroenterostomy (BEE) have been proposed to help mitigate this complication. BEE technique was first described by Heinrich Braun in the late nineteenth century with the intent of diminishing alkaline reflux gastritis and bilious emesis during gastric surgery as well as decompressing the afferent jejunal limb [11, 12]. This technique involves a side-to-side jejunojejunostomy created between the afferent and efferent limb just distal to the gastrojejunostomy. The application of BEE theoretically improves functional outflow from the gastrojejunostomy by creating a low-pressure route that reduces luminal stasis and has therefore been postulated to reduce the incidence of DGE following PD [13]. Notably, existing investigations yielded conflicting results. For instance, Xu et al. [14] reported a reduced incidence of DGE with the addition of BEE whereas Fujieda et al. [15] found no difference and suggested that diversion of biliary flow alone may be insufficient to produce a clinically meaningful effect.

With the lack of consensus regarding the role of BEE in PD and the emergence of several recent randomized controlled trials (RCTs) and high-quality observational studies, an updated systematic review and meta-analysis was warranted to provide a comprehensive synthesis of the literature to date. Thus, this meta-analysis aims to evaluate whether adjunctive BEE reduces the incidence and severity of DGE as well as postoperative morbidity after PD.

## Methods and materials

### Data resources and search strategy

A comprehensive literature search was performed across several electronic databases, including CINAHL, PubMed, Embase, Scopus, Web of Science and Cochrane Library, from inception until November 4, 2025. This systematic review was reported in compliance with the Preferred Reporting Items for Systematic Reviews and Meta-analysis (PRISMA) reporting guidelines [16]. The literature search strategy was designed and conducted by an experienced librarian in collaboration with the study’s principal investigator. Controlled vocabulary supplemented with keywords was used to identify studies involving adult patients undergoing PD with BEE. The bibliography of retrieved papers was further manually searched for additional studies. A detailed description of all search terms is available in Supplementary Item 1. This review was prospectively registered with PROSPERO (CRD420251160291).

### Eligibility criteria

Eligible studies were RCTs and cohort studies that fulfilled the following inclusion criteria: (1) adult patients ≥ 18 years of age who underwent PD for any indication; (2) compared patients undergoing PD with and without BEE; (3) reported on at least one of the primary outcomes, including the overall incidence of DGE and postoperative morbidity. In cases where studies originated from the same institution and/or authors, the most recent study or that of higher quality was selected for inclusion. Case reports, case series, abstracts, reviews, and articles that were not reported in English were excluded. Additionally, this review excluded studies involving cases in which invasive operations were not limited to PD with or without BEE.

### Screening and risk of bias assessment

Two out of six independent assessors (AG, GA, KM, LMD, MA, SB) conducted individual article screening and data extraction. Two of six authors (GA, KM, LMD, MA, MYH, SB) assessed bias using the risk of bias in non-randomized studies of Interventions (ROBINS-I) and risk of bias tool for randomized trials (ROB-2) tools [17, 18]. Any disagreements were discussed with co-authors and adjudicated by HN. The certainty of evidence regarding DGE and morbidity analyses was evaluated using the Grading of Recommendations, Assessment, Development, and Evaluation (GRADE) approach, with a priori thresholds set for small, moderate, and large effects [19].

### Extracted outcomes

This analysis aimed to evaluate the outcomes of BEE as an adjunct to PD. The baseline clinical characteristics extracted were as follows: body mass index (BMI), indication for PD, PD type (i.e., standard or modified), route of reconstruction (i.e., antecolic or retrocolic), BEE distance from gastrojejunostomy (GJ), BEE anastomotic orifice size, operative approach, and pancreatic texture. Intraoperative outcomes included operative time, hospital length of stay (LOS), estimated blood loss (EBL), and intraoperative blood transfusion rates. Postoperative outcomes of interest included rates of DGE, postoperative pancreatic fistula (POPF), overall serious morbidity (Clavien-Dindo grade ≥ III) [20], reoperation, the indication for reoperation, short-term mortality (< 30 days), and overall mortality.

### Braun enteroenterostomy selection criteria and definitions

The decision to perform BEE was randomized across five studies [15, 21–24], based on surgeon preference across two studies [10, 11], and performed in all consecutive cases during the later study period across three studies [14, 25, 26]. Across fourteen studies [10, 11, 14, 15, 21, 23–31], DGE was classified into three grades of increasing severity according to the International Study Group for Pancreatic Surgery (ISGPS) definition [32]. One study defined DGE as “gastric stasis requiring a nasogastric tube (NGT) for ≥10 days or the inability to tolerate a regular diet for 14 days after the operation” [22]. Similarly, across twelve studies [10, 14, 21, 23–31], POPF was defined based on the ISGPS grading system [33]. For the purposes of this study, clinically relevant DGE and POPF were defined as grades B and C combined.

### Statistical analysis

A random-effects method was used to analyze the pooled means and proportions of our data. Continuous and dichotomous data were analyzed using the generic inverse variance method and the Mantel–Haenszel method, respectively. To directly compare patients undergoing PD with and without BEE, studies that reported outcomes of both treatments were assessed (two-arm analysis). The heterogeneity of effect size estimates across the studies was quantified using the *Q* statistic and *I*^2^. An *I*^2^ value of 0–25% indicates insignificant statistical heterogeneity, 26–50% indicates low heterogeneity, and 51–100% indicates high heterogeneity [34]. A leave-one-out sensitivity analysis was performed to evaluate the influence of individual studies on the pooled estimate by sequentially excluding each study and recalculating the combined effect estimates from the remaining studies. If mean and standard deviation (SD) were unavailable, the median was converted to mean using formulas from the Cochrane Handbook for Systematic Reviews of Interventions [35]. Data analysis was conducted using Review Manager (RevMan) software, version 5.4 (The Cochrane Collaboration, Copenhagen, Denmark, 2020).

## Results

### Study selection and patient characteristics

The initial search identified a total of 340 relevant articles, of which 65 full-text studies were assessed for eligibility. Ultimately, seventeen studies [10, 11, 14, 15, 21–31, 36, 37] including 2731 patients were included for analysis, comprising 1407 (51.5%) patients with BEE and 1324 (48.5%) without BEE. Ten studies [10, 11, 14, 24–26, 28, 31, 36, 37] were retrospective in design, four [15, 21–23] were RCTs, while two [27, 30] were prospective studies with retrospective control groups and one [29] was a prospective cohort study. One study [37] was conducted in a multicenter setting, while the remainder were conducted in a single institution. The mean age across studies ranged from 54.7 to 70.0 years in patients with BEE and from 52.5 to 72.0 years in patients without BEE. In patients with BEE, 574 (40.8%) patients were female, while 547 (41.3%) were female in patients without BEE. The pooled mean BMI did not differ between groups (MD = − 0.11 kg/m^2^, 95% CI − 0.67, 0.45, *I*^2^ = 43%) across eight studies [10, 15, 25–28, 30, 31]. A PRISMA flowchart of the study selection process is depicted in Supplementary Item 2. The baseline characteristics of the included studies are described in Table 1.

**Table 1:** Baseline characteristics of included studies

Study	Publication year	Country	Study type	Number of centers	Data collection years	Number of patients (*n*)	BEE group, *n* (%)	NB group, *n* (%)	Female sex, *n* (%)	Age, mean ± SD (Years)
Carmel et al	2025	Israel	Retrospective	Single center	December 2014–February 2021	214	76 (35.5)	138 (64.5)	BEE: 36 (47.4) NB: 74 (53.6)	BEE: 66.0 ± 11.0 NB: 65.0 ± 11.0
Cordesmeyer et al	2014	Germany	Retrospective	Single center	September 2004–December 2011	113	51 (45.1)	62 (54.9)	BEE: 24 (47.1) NB: 30 (48.4)	BEE: 66.0 ± 12.9 NB: 66.8 ± 11.6
Fujieda et al	2017	Japan	RCT	Single center	August 2011–February 2016	68	34 (50.0)	34 (50.0)	BEE: 14 (41.2) NB: 10 (29.4)	BEE: 66.0 ± 5.8 NB: 72.0 ± 9.5
Hochwald et al	2010	United States	Retrospective	Single center	2001–2006	105	70 (66.7)	35 (33.3)	BEE: NR NB: NR	BEE: 65.0 ± NR NB: 64.0 ± NR
Hwang et al	2016	Korea	RCT	Single center	February 2013–June 2014	60	30 (50.0)	30 (50.0)	BEE: 11 (36.7) NB: 11 (36.7))	BEE: 69.0 ± 8.0 NB: 63.0 ± 9.0
Kakaei et al	2015	Iran	RCT	Single center	June 2013–December 2013	30	15 (50.0)	15 (50.0)	BEE: 5 (33.3) NB: 5 (33.3)	BEE: 57.3 ± 13.8 NB: 55.3 ± 13.2
Luu et al	2019	Germany	Retrospective	Single center	NR	100	50 (50.0)	50 (50.0)	BEE: 33 (66.0) NB: 33 (66.0)	BEE: 63.0 ± 13.0 NB: 65.0 ± 11.0
Marimuthu et al	2025	India	Prospective with retrospective controls	Single center	May 2023–August 2024	96	24 (25.0)	72 (75.0)	BEE: 14 (58.3) NB: 23 (31.9)	BEE: 54.7 ± 12.3 NB: 52.5 ± 12.5
Meng et al	2015	China	Retrospective	Single center	January 2009–March 2014	203	98 (48.3)	105 (51.7)	BEE: 41 (41.8) NB: 37 (35.2)	BEE: 62.0 ± NR NB: 60.3 ± NR
Nageswaran et al	2015	United Kingdom	Retrospective	Multicenter	NR	300	83 (27.7)	217 (72.3)	BEE: 36 (43.4) NB: 95 (43.8)	BEE: 67.0 ± 12.8 NB: 70.0 ± 18.5
Nikfarjam et al	2012	Australia	Retrospective	Single center	August 2009–November 2011	44	24 (54.5)	20 (45.5)	BEE: 9 (37.5) NB: 6 (30)	BEE: 67.0 ± 9.0 NB: 70.0 ± 8.5
Tanaka et al	2022	Japan	Prospective	Single center	August 2010–November 2015	145	51 (35.2)	94 (64.8)	BEE: 19 (37.3) NB: 39 (41.5)	BEE: 70.0 ± 14.0 NB: 70.0 ± 11.3
Usenko et al	2023	Ukraine	Prospective with retrospective controls	Single center	2015–2023	162	102 (63.0)	60 (37.0)	BEE: 40 (39.2) NB: 25 (41.7)	BEE: 59.6 ± 9.9 NB: 55.7 ± 11.6
Vutukuru et al	2019	India	RCT	Single center	June 2012–July 2016	104	48 (46.2)	56 (53.8)	BEE: 20 (41.7) NB: 20 (35.7)	BEE: 55.2 ± 9.9 NB: 52.6 ± 10.3
Watanabe et al	2015	Japan	Retrospective	Single center	January 2008–June 2013	185	98 (53.0)	87 (47.0)	BEE: 41 (41.8) NB: 40 (46.0)	BEE: 67.0 ± 15.8 NB: 70.0 ± 16.0
Xu et al	2015	China	Retrospective	Single center	June 2000–March 2013	407	206 (50.6)	201 (49.4)	BEE: 82 (39.8) NB: 73 (36.3)	BEE: 57.9 ± 10.6 NB: 58.1 ± 11.3
Zhang et al	2014	China	Retrospective	Single center	January 2009–May 2013	395	347 (87.8)	48 (12.2)	BEE: 149 (42.9) NB: 26 (54.2)	BEE: 57.0 ± 11.2 NB: 58.0 ± 19.4

*BEE* Braun enteroenterostomy, *NB* Non-Braun enteroenterostomy, *NR* Not reported, *RCT* Randomized controlled trial, *SD* Standard deviation

### Risk of bias assessment and certainty of evidence

Among the thirteen observational studies assessed using the ROBINS-I tool, six studies [10, 14, 26–28, 31] were deemed to have moderate risk of bias with potential confounding and selection bias. Seven studies [11, 24, 25, 29, 30, 36, 37] were determined to have serious risk of bias due to similar reasons, namely lack of adjusting for confounding factors and concerns regarding the selection of participants. Among the remaining four RCTs, using the ROB-2 tool, three studies [15, 22, 23] were deemed to have low risk of bias. In the remaining one study [21], some concerns were present regarding the randomization process. Nonetheless, all the studies included were deemed adequate within the selection domain with adequate ascertainment of exposures and outcomes. The results of the risk of bias assessment and certainty of evidence through a GRADE evidence table are illustrated in Supplementary Items 3 and 4.

### Clinical characteristics

Across seven studies [10, 21, 23, 27, 29–31], PD was performed via an open approach (*n* = 419, 97.7%) or laparoscopic approach (*n* = 10, 2.3%) in patients with BEE, while in patients without BEE, 513 (95.5%) were performed via an open and 24 (4.5%) via a laparoscopic approach, respectively. In patients with BEE, classic PD was performed in 1,031 cases (73.3%), pylorus-preserving PD (PPPD) in 311 cases (22.1%), and subtotal stomach-preserving PD (SSPPD) in 65 cases (4.6%). In patients without BEE, classic PD was performed in 853 cases (64.4%), PPPD in 381 cases (28.8%), and SSPPD in 90 cases (6.8%). Across ten studies [10, 11, 14, 15, 23, 25–29], an antecolic reconstruction approach was used in 659 (96.6%) cases while a retrocolic approach was used in 23 (3.4%) cases undergoing PD with BEE. In patients without BEE, an antecolic reconstruction approach was used in 755 (92.4%) cases while a retrocolic approach was used in 62 (7.6%). Across fourteen studies [10, 11, 15, 21–24, 26–31, 36], the indication for PD in patients with BEE was malignancy across 617 (57.8%) patients, benign in 401 (37.6%), and unspecified in 49 (4.6%) cases. In patients without BEE, the indication was malignant in 602 (78.0%) patients, benign in 139 (18.0%), and unspecified in 31 (4.0%) cases. The pancreatic texture was described as soft in 371 (54.8%) BEE cases and 155 (50.8%) non-BEE cases across seven studies [15, 23, 24, 26, 27, 30, 31]. The detailed clinical characteristics across the two groups are described in Table 2.

**Table 2:** Clinical characteristics of included studies

Study	BEE distance from GJ (cm)	BEE anastomotic orifice (cm)	Surgical approach, *n* (%)		Surgical procedure, *n* (%)			Reconstruction route, *n* (%)		Indication, *n* (%)			Soft pancreas, *n* (%)
			Open	Laparoscopic	PD	PPPD	SSPPD	Antecolic	Retrocolic	Malignant	Benign	Unspecified	
Carmel et al	20	3	BEE: 76 (100) NB: 138 (100)	BEE: 0 (0) NB: 0 (0)	BEE: 76 (100) NB: 138 (100)	BEE: 0 (0) NB: 0 (0)	BEE: 0 (0) NB: 0 (0)	BEE: 76 (100) NB: 138 (100)	BEE: 0 (0) NB: 0 (0)	BEE: 67 (88.2) NB: 115 (83.3)	BEE: 9 (11.8) NB: 23 (16.7)	BEE: 0 (0) NB: 0 (0)	BEE: NR NB: NR
Cordesmeyer et al	10–15 *	NR	BEE: NR NB: NR	BEE: NR NB: NR	BEE: 0 (0) NB: 0 (0)	BEE: 51 (100) NB: 62 (100)	BEE: 0 (0) NB: 0 (0)	BEE: 28 (54.9) NB: 0 (0)	BEE: 23 (45.1) NB: 62 (100)	BEE: 0 (0) NB: 0 (0)	BEE: NR NB: NR	BEE: NR NB: NR	BEE: NR NB: NR
Fujieda et al	20	3	BEE: NR NB: NR	BEE: NR NB: NR	BEE: 0 (0) NB: 0 (0)	BEE: 0 (0) NB: 0 (0)	BEE: 34 (100) NB: 34 (100)	BEE: 34 (100) NB: 34 (100)	BEE: 0 (0) NB: 0 (0)	BEE: 28 (82.4) NB: 26 (76.5)	BEE: 6 (17.6) NB: 8 (23.5)	BEE: 0 (0) NB: 0 (0)	BEE: 19 (55.9) NB: 20 (58.8)
Hochwald et al	25	NR	BEE: NR NB: NR	BEE: NR NB: NR	BEE: 70 (100) NB: 35 (100)	BEE: 0 (0) NB: 0 (0)	BEE: 0 (0) NB: 0 (0)	BEE: 70 (100) NB: 35 (100)	BEE: 0 (0) NB: 0 (0)	BEE: 48 (68.6) NB: 21 (60)	BEE: 7 (10) NB: 2 (5.7)	BEE: 15 (21.4) NB: 12 (34.3)	BEE: NR NB: NR
Hwang et al	30*	NR	BEE: 30 (100) NB: 30 (100)	BEE: 0 (0) NB: 0 (0)	BEE: 0 (0) NB: 0 (0)	BEE: 30 (100) NB: 30 (100)	BEE: 0 (0) NB: 0 (0)	BEE: NR NB: NR	BEE: NR NB: NR	BEE: 26 (86.7) NB: 28 (93.3)	BEE: 4 (13.3) NB: NR	BEE: 0 (0) NB: 2 (6.7)	BEE: NR NB: NR
Kakaei et al	45	NR	BEE: NR NB: NR	BEE: NR NB: NR	BEE: 15 (100) NB: 15 (100)	BEE: 0 (0) NB: 0 (0)	BEE: 0 (0) NB: 0 (0)	BEE: NR NB: NR	BEE: NR NB: NR	BEE: 12 (80) NB: 12 (80)	BEE: NR NB: NR	BEE: 3 (20) NB: 3 (20)	BEE: NR NB: NR
Luu et al	NR	NR	BEE: NR NB: NR	BEE: NR NB: NR	BEE: 0 (0) NB: 0 (0)	BEE: 50 (100) NB: 50 (100)	BEE: 0 (0) NB: 0 (0)	BEE: NR NB: NR	BEE: NR NB: NR	BEE: 33 (66.7) NB: 33 (66.7)	BEE: 17 (33.3) NB: 17 (33.3)	BEE: 0 (0) NB: 0 (0)	BEE: NR NB: NR
Marimuthu et al	NR	NR	BEE: 24 (100) NB: 72 (100)	BEE: 0 (0) NB: 0 (0)	BEE: 24 (100) NB: 72 (100)	BEE: 0 (0) NB: 0 (0)	BEE: 0 (0) NB: 0 (0)	BEE: 24 (100) NB: 72 (100)	BEE: 0 (0) NB: 0 (0)	BEE: 21 (87.5) NB: 0 (0)	BEE: NR NB: NR	BEE: 3 (12.5) NB: NR	BEE: 12 (50.0) NB: NR
Meng et al	NR	NR	BEE: NR NB: NR	BEE: NR NB: NR	BEE: 98 (100) NB: 105 (100)	BEE: 0 (0) NB: 0 (0)	BEE: 0 (0) NB: 0 (0)	BEE: 98 (100) NB: 105 (100)	BEE: 0 (0) NB: 0 (0)	BEE: 89 (90.8) NB: 92 (87.6)	BEE: 8 (8.2) NB: 9 (8.6)	BEE: 1 (1.0) NB: 4 (3.8)	BEE: NR NB: NR
Nageswaran et al	NR	NR	BEE: NR NB: NR	BEE: NR NB: NR	BEE: 17 (20.5) NB: 96 (44.2)	BEE: 66 (79.5) NB: 121 (55.8)	BEE: 0 (0) NB: 0 (0)	BEE: NR NB: NR	BEE: NR NB: NR	BEE: 0 (0) NB: 0 (0)	BEE: NR NB: NR	BEE: NR NB: NR	BEE: NR NB: NR
Nikfarjam et al	25	NR	BEE: NR NB: NR	BEE: NR NB: NR	BEE: 24 (100) NB: 20 (100)	BEE: 0 (0) NB: 0 (0)	BEE: 0 (0) NB: 0 (0)	BEE: 24 (100) NB: 20 (100)	BEE: 0 (0) NB: 0 (0)	BEE: 18 (75) NB: 18 (90)	BEE: 6 (25) NB: 2 (10)	BEE: 0 (0) NB: 0 (0)	BEE: 12 (50.0) NB: 9 (45.0)
Tanaka et al	NR	NR	BEE: 41 (80.4) NB: 70 (74.5)	BEE: 10 (19.6) NB: 24 (25.5)	BEE: 4 (7.8) NB: 7 (7.4)	BEE: 16 (31.4) NB: 31 (33.0)	BEE: 31 (60.8) NB: 56 (59.6)	BEE: 51 (100) NB: 94 (100)	BEE: 0 (0) NB: 0 (0)	BEE: 42 (82.4) NB: 76 (80.9)	BEE: 7 (13.7) NB: 16 (17.0)	BEE: 2 (3.9) NB: 2 (2.1)	BEE: NR NB: NR
Usenko et al	15	1–1.5	BEE: 102 (100) NB: 60 (100)	BEE: 0 (0) NB: 0 (0)	BEE: 102 (100) NB: 60 (100)	BEE: 0 (0) NB: 0 (0)	BEE: 0 (0) NB: 0 (0)	BEE: NR NB: NR	BEE: NR NB: NR	BEE: 100 (98.0) NB: 56 (93.3)	BEE: 2 (2.0) NB: 4 (6.7)	BEE: 0 (0) NB: 0 (0)	BEE: 59 (57.8) NB: 33 (55.0)
Vutukuru et al	25	4	BEE: 48 (100) NB: 56 (100)	BEE: 0 (0) NB: 0 (0)	BEE: 48 (100) NB: 56 (100)	BEE: 0 (0) NB: 0 (0)	BEE: 0 (0) NB: 0 (0)	BEE: 48 (100) NB: 56 (100)	BEE: 0 (0) NB: 0 (0)	BEE: 44 (91.7) NB: 54 (96.4)	BEE: 0 (0) NB: 0 (0)	BEE: 4 (8.3) NB: 2 (3.6)	BEE: 22 (45.8) NB: 28 (50.0)
Watanabe et al	10*	NR	BEE: 98 (100) NB: 87 (100)	BEE: 0 (0) NB: 0 (0)	BEE: 0 (0) NB: 0 (0)	BEE: 98 (100) NB: 87 (100)	BEE: 0 (0) NB: 0 (0)	BEE: NR NB: NR	BEE: NR NB: NR	BEE: 74 (75.5) NB: 67 (77.0)	BEE: 21 (21.4) NB: 16 (18.4)	BEE: 3 (3.1) NB: 4 (4.6)	BEE: 53 (54.1) NB: 41 (47.1)
Xu et al	NR	NR	BEE: NR NB: NR	BEE: NR NB: NR	BEE: 206 (100) NB: 201 (100)	BEE: 0 (0) NB: 0 (0)	BEE: 0 (0) NB: 0 (0)	BEE: 206 (100) NB: 201 (100)	BEE: 0 (0) NB: 0 (0)	BEE: 0 (0) NB: 0 (0)	BEE: NR NB: NR	BEE: NR NB: NR	BEE: NR NB: NR
Zhang et al	10–20	NR	BEE: NR NB: NR	BEE: NR NB: NR	BEE: 347 (100) NB: 48 (100)	BEE: 0 (0) NB: 0 (0)	BEE: 0 (0) NB: 0 (0)	BEE: NR NB: NR	BEE: NR NB: NR	BEE: 15 (4.3) NB: 4 (8.3)	BEE: 314 (90.5) NB: 42 (87.5)	BEE: 18 (5.2) NB: 2 (4.2)	BEE: 194 (55.9) NB: 24 (50.0)

*Distance from DJ

*BEE* Braun enteroenterostomy, *DJ* Duodenojejunostomy, *GJ* Gastrojejunostomy, *NB* Non-Braun enteroenterostomy, *NR* Not reported, *PD* Pancreatoduodenectomy, *PPPD* Pylorus-preserving pancreatoduodenectomy, *RCT* Randomized controlled trial, *SD* Standard deviation, *SSPPD* Subtotal stomach-preserving pancreatoduodenectomy

### Perioperative outcomes

**Fig. 1:**
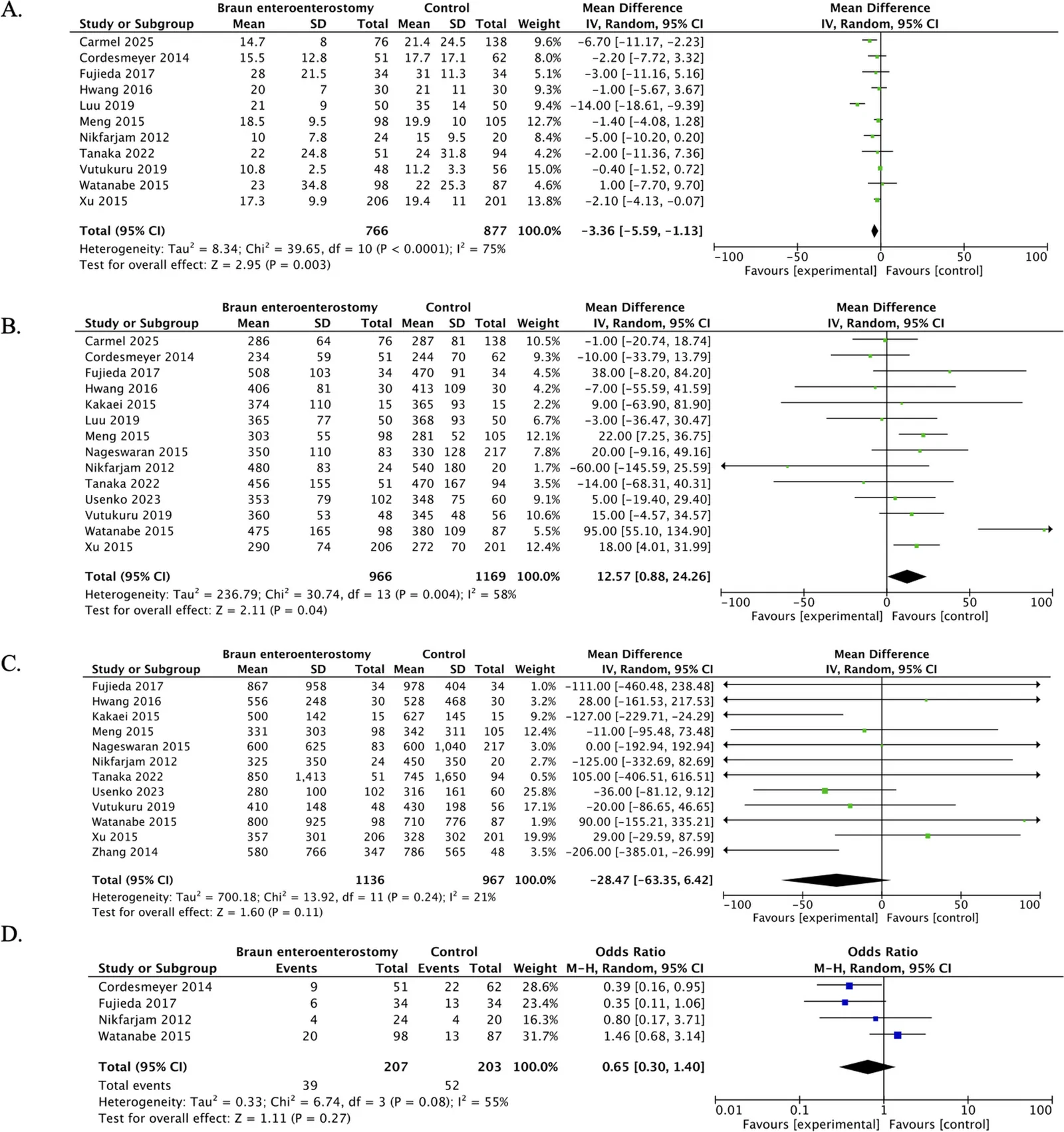
The pooled estimate of perioperative outcomes: **A**. hospital stay, **B**. operative time, **C**. estimated blood loss, **D**. intraoperative blood transfusion

Hospital LOS was reported in eleven studies [10, 14, 15, 21, 23, 25, 26, 28, 29, 31, 36], where BEE was associated with a lower LOS (MD = − 3.4 days, 95% CI − 5.6, − 1.1, *I*^2^ = 75%). Across fourteen studies [10, 14, 15, 21–23, 25, 26, 28–31, 36, 37], operative time was longer in patients undergoing PD with BEE (MD = 12.6 min, 95% CI 0.9, 24.3, *I*^2^ = 58%). EBL was reported across twelve studies [14, 15, 21–24, 26, 28–31, 37], with both groups achieving similar results (MD = − 28.5, 95% CI − 63.4, 6.4, *I*^2^ = 21%). Intraoperative transfusion rates were reported across four studies [15, 25, 26, 31], achieving similar results between groups (OR = 0.65, 95% CI 0.30, 1.40, *I*^2^ = 55%). The perioperative outcomes of the included studies are detailed in Fig. 1.

### Postoperative complications

**Fig. 2:**
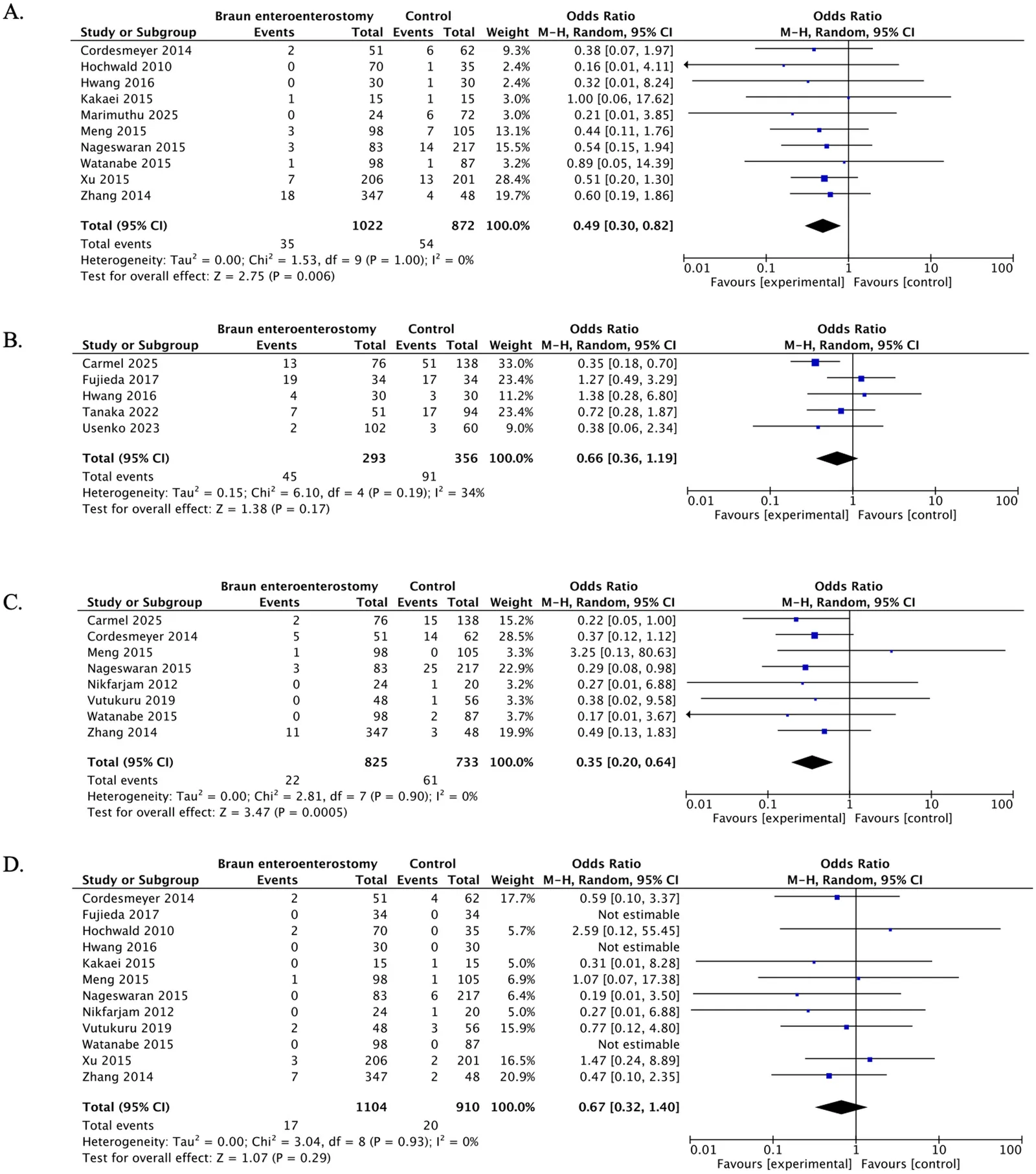

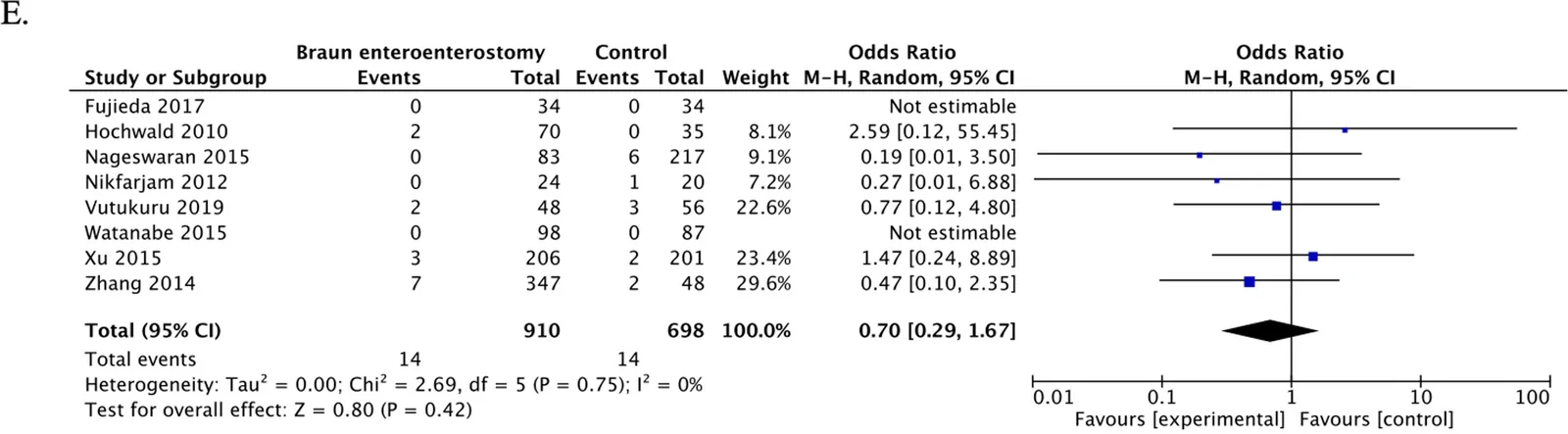
The pooled estimate of postoperative complications: **A**. bile leak, **B**. serious morbidity (Clavien–Dindo grade ≥ III), **C**. reoperation, **D**. overall mortality, **E**. short-term mortality (< 30 days)

Across ten studies [11, 14, 21, 22, 24, 25, 27, 28, 31, 37], BEE was associated with a lower rate of bile leak (OR = 0.49, 95% CI 0.30, 0.82, *I*^2^ = 0%). Serious morbidity was reported in five studies [10, 15, 21, 29, 30], with similar results between groups (OR = 0.66, 95% CI 0.36, 1.19, *I*^2^ = 34%). Across eight studies [10, 23–26, 28, 31, 37], the rate of reoperation was lower in patients with BEE (OR = 0.35, 95% CI 0.20, 0.64, *I*^2^ = 0%). Across twelve studies [11, 14, 15, 21–26, 28, 31, 37], no difference was found in overall mortality between groups (OR = 0.67, 95% CI 0.32, 1.40, *I*^2^ = 0%). Similarly, across eight studies [11, 14, 15, 23, 24, 26, 31, 37], there was no difference in short-term mortality between the two groups (OR = 0.70, 95% CI 0.29, 1.67, *I*^2^ = 0%). The postoperative complications of the included studies are summarized in Fig. 2.

### DGE and POPF grades

**Fig. 3:**
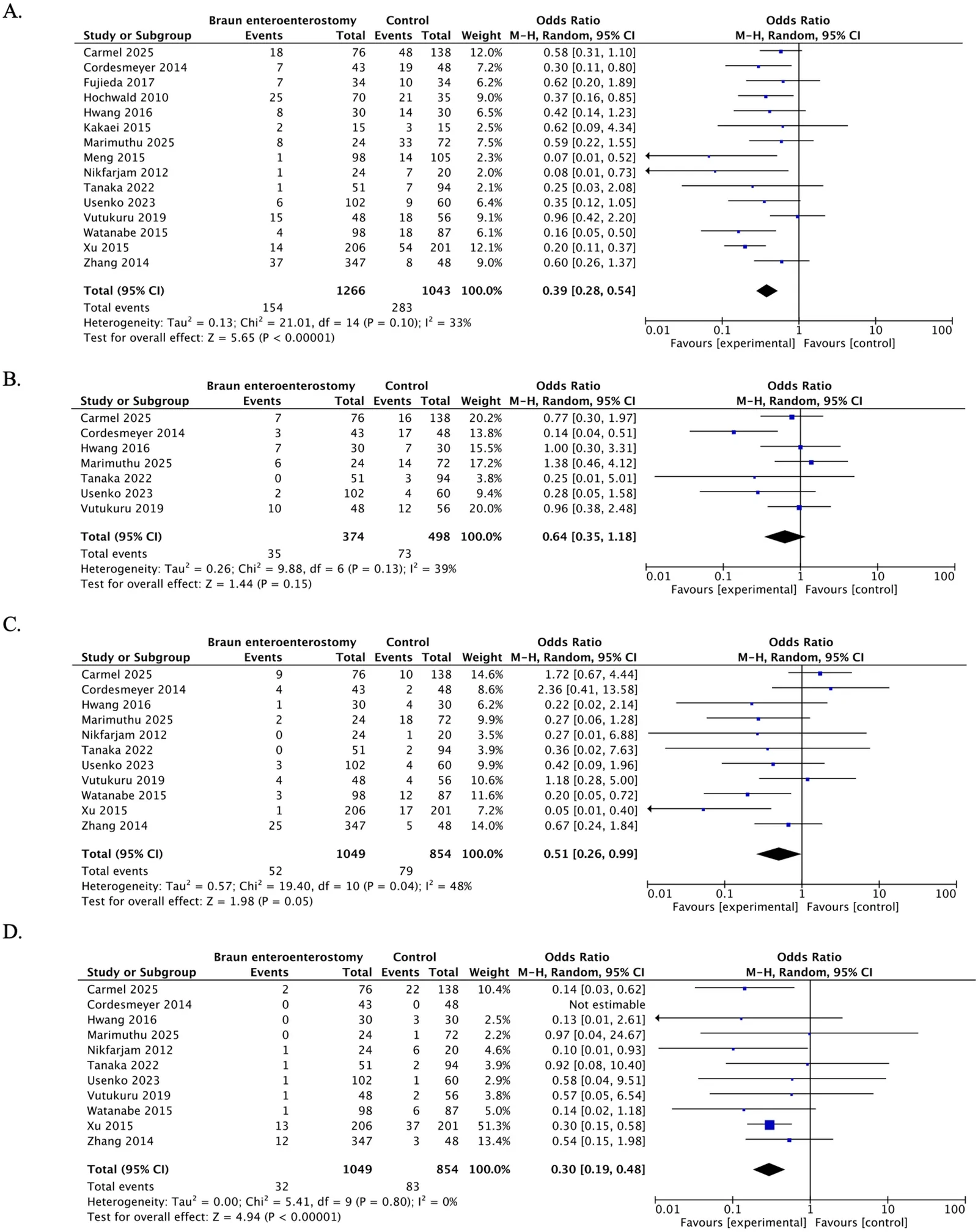

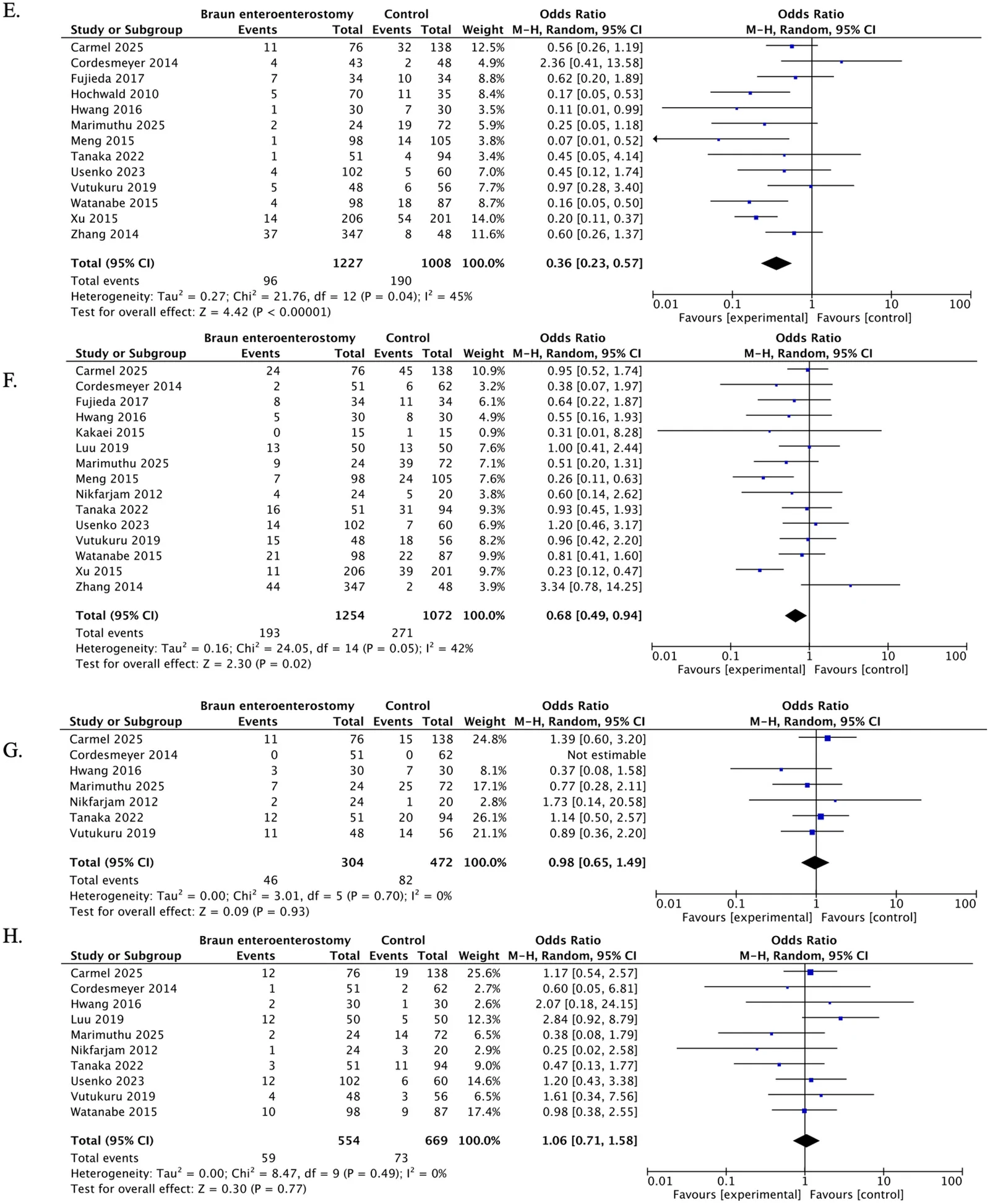

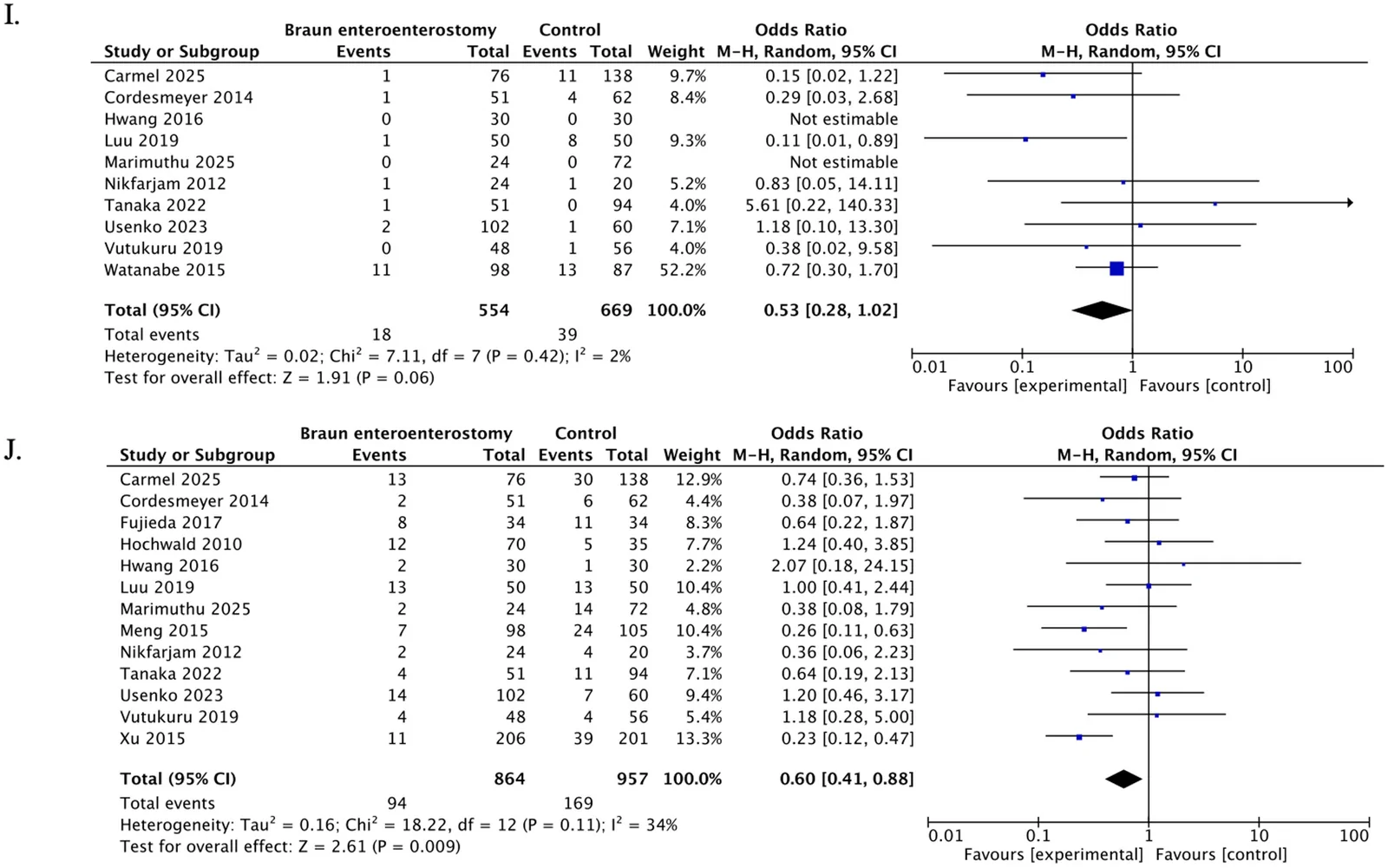
The pooled estimate of DGE and POPF rates: **A**. overall DGE, **B**. DGE A, **C**. DGE B, **D**. DGE C, **E**. clinically relevant DGE, **F**. overall POPF, **G**. biochemical leak, **H**. POPF B, **I**. POPF C, **J**. clinically relevant POPF

The overall rate of DGE was reported in fifteen studies [10, 11, 14, 15, 21–31], with BEE having a lower rate of DGE (OR = 0.39, 95% CI 0.28, 0.54, *I*^2^ = 33%). Pooled analysis of Grade A DGE across seven studies [10, 21, 23, 25, 27, 29, 30] showed no difference between groups (OR = 0.64, 95% CI 0.35, 1.18, *I*^2^ = 39%). Grade B DGE was reported across eleven studies [10, 14, 21, 23–27, 29–31] and was lower in patients with BEE (OR = 0.51, 95% CI 0.26, 0.99, *I*^2^ = 48%). Similarly, Grade C DGE was reported in eleven studies [10, 14, 21, 23–27, 29–31] and was lower in patients with BEE (OR = 0.30, 95% CI 0.19, 0.48, *I*^2^ = 0%). Clinically relevant DGE, as reported in thirteen studies [10, 11, 14, 15, 21, 23–25, 27–31], was lower in patients with BEE (OR = 0.36, 95% CI 0.23, 0.57, *I*^2^ = 45%). Pooled analysis of overall POPF across fifteen studies [10, 14, 15, 21–31, 36] revealed a lower rate in patients with BEE (OR = 0.68, 95% CI 0.49, 0.94, *I*^2^ = 42%). Biochemical leak was reported in seven studies [10, 21, 23, 25–27, 29], with no difference found between groups (OR = 0.98, 95% CI 0.65, 1.49, *I*^2^ = 0%). Grade B POPF was reported in ten studies [10, 21, 23, 25–27, 29–31, 36] with both groups having similar rates (OR = 1.06, 95% CI 0.71, 1.58, *I*^2^ = 0%). Across ten studies [10, 21, 23, 25–27, 29–31, 36], no difference was found in the rate of Grade C POPF (OR = 0.53, 95% CI 0.28, 1.02, *I*^2^ = 2%). Across thirteen studies [10, 11, 14, 15, 21, 23, 25–30, 36], the rate of clinically relevant POPF was lower in patients with BEE (OR = 0.60, 95% CI 0.41, 0.88, *I*^2^ = 34%). The detailed forest plots of DGE and POPF rates are presented in Fig. 3.

In the leave-one-out sensitivity analysis, the pooled estimates for overall DGE (OR range: 0.36–0.44), clinically relevant DGE (OR range: 0.33–0.40), and clinically relevant POPF (OR range: 0.56–0.70) remained significant following the exclusion of each individual study. The overall POPF estimate remained significant after all but two single-study exclusions (OR range: 0.64–0.70), losing statistical significance upon exclusion of either Meng et al. [28] (OR = 0.74, 95% CI 0.54, 1.01) or Xu et al. [14] (OR = 0.78, 95% CI 0.60, 1.01). Combined exclusion of the two most heavily weighted retrospective cohorts, Xu et al. [14] and Zhang et al. [24], did not alter the direction or significance of any estimate (overall DGE: OR = 0.42, 95% CI 0.30, 0.59; clinically relevant DGE: OR = 0.38, 95% CI 0.22, 0.64; overall POPF: OR = 0.75, 95% CI 0.58, 0.96, *I*^2^ = 0%; clinically relevant POPF: OR = 0.70, 95% CI 0.50, 0.96). The complete results are presented in Supplementary Item 5.

### Subgroup analysis including RCTs only

A subgroup analysis was conducted, focusing on data from the included RCTs. Across four studies [15, 21–23] including 262 patients, 127 (48.5%) patients underwent PD with BEE and 135 (51.5%) without BEE. The mean age across studies ranged from 55.2 to 69.0 years in patients with BEE and from 52.6 to 70.0 years in patients without BEE. In patients with BEE, 50 (39.4%) patients were female, while 46 (34.1%) were female in patients without BEE. There were no differences found in operative time (MD = 15.1 min, 95% CI − 1.4, 31.6, *I*^2^ = 0%) and EBL (MD = − 51.1 mL, 95% CI − 117.6, 15.5, *I*^2^ = 19%) between groups. Across three studies [15, 21, 23], there was no difference in hospital LOS between groups (MD = − 0.5 days, 95% CI − 1.6, 0.6, *I*^2^ = 0%). The overall rate of DGE was similar between groups (OR = 0.68, 95% CI 0.39, 1.17, *I*^2^ = 0%). Clinically relevant DGE, as reported in three studies [15, 21, 23], was no different between groups (OR = 0.55, 95% CI 0.21, 1.47, *I*^2^ = 31%). Pooled analysis of overall POPF revealed no difference between groups (OR = 0.74, 95% CI 0.42, 1.31, *I*^2^ = 0%). Across three studies [15, 21, 23], the rate of clinically relevant POPF was similar between groups (OR = 0.89, 95% CI 0.39, 1.99, *I*^2^ = 0%). The detailed forest plots of the subgroup analyses are presented in Supplementary Items 6.

## Discussion

PD remains the standard surgical treatment for benign and malignant disease of the pancreatic head and periampullary region [38]. Despite substantial reduction in perioperative mortality rates in contemporary practice, postoperative morbidity continues to be a challenge associated with reconstruction-related complications [27]. The primary aim of this meta-analysis was to evaluate the safety and efficacy of BEE compared with standard reconstruction in patients undergoing PD. This meta-analysis demonstrates that the addition of BEE to standard PD reconstruction is associated with lower rates of overall and clinically relevant DGE, clinically relevant POPF, bile leak, and reoperation, as well as a shorter LOS. Serious morbidity and mortality did not differ between groups, and no complication attributable directly to BEE was reported in any included study.

The present meta-analysis is consistent with prior literature, demonstrating that BEE is associated with a reduction in clinically relevant DGE following PD [13, 14, 39]. This is particularly relevant considering that DGE remains the most common postoperative complication after PD, affecting a substantial proportion of patients [11]. Although DGE is rarely life-threatening, it impairs recovery by delaying nutritional recovery, increasing reliance on total parenteral nutrition and worsening postoperative nutritional status [21]. DGE is further associated with prolonged length of stay and reduced postoperative quality of life [15]. The precise pathophysiology underlying DGE following PD remains uncertain and is multifactorial, with many proposed mechanisms. Bile reflux appears to be most likely influenced by BEE addition as it diverts biliopancreatic secretions away from the gastrojejunostomy by providing an alternative low-resistance pathway. This facilitates the flow of enteric contents, limiting bile reflux into the stomach and reducing mucosal irritation [11]. However, this is challenged by an RCT by Fujieda et al., employing 24-h intragastric bilirubin monitoring, which found that BEE did not reduce intragastric bile reflux and had only minor impact on DGE incidence [15]. Beyond the diversion of bile reflux, the literature proposes several alternative mechanisms for DGE involving mechanical, inflammatory, and physiological disruptions. It has been hypothesized that BEE provides essential mechanical stabilization by straightening and securing the jejunal limbs, which prevents the gastroenterostomy from twisting [26, 31]. This structural benefit is complemented by the dual exit route theory, supporting that BEE facilitates gastric emptying by allowing contents to flow in both directions, bypassing functional atony or early postoperative edema [11]. Collectively, these findings suggest that DGE is likely attributed to many factors, and an individualized approach is required to manage each cause. The effect of BEE may also be context-dependent, potentially modulated by reconstruction route, or loop length, although the lack of stratified reporting precluded a formal analysis of these factors [15, 25]. Notably, the same rationale underlies the use of BEE after distal gastrectomy with Billroth II reconstruction, where it is performed primarily to divert duodenal content away from the gastric remnant and to decompress the afferent limb, thereby limiting alkaline reflux gastritis, bilious emesis and afferent loop syndrome [11, 12]. The mechanistic emphasis differs after PD, however, because the afferent limb additionally carries the pancreaticojejunal and hepaticojejunal anastomoses, so decompression is directed at the biliopancreatic anastomoses as well as at the gastrojejunostomy, and the reconstruction route itself influences DGE in this setting [4]. This difference in purpose may explain why the gastrectomy literature is oriented toward long-term symptomatic endpoints whereas the PD literature is centered on DGE and POPF and cautions against direct extrapolation between the two settings.

The effect of BEE appeared confined to the clinically relevant grades of DGE, with no difference in Grade A DGE between groups. This distribution is physiologically plausible as Grade A DGE reflects the transient gastric stasis that commonly follows PD irrespective of reconstruction and resolves with routine perioperative care, whereas the mechanisms attributed to BEE likely act on the sustained afferent limb loading and bile-induced mucosal irritation that prolong stasis beyond the first postoperative week. As such, BEE appears to not prevent DGE from occurring but limit its progression to a clinically relevant grade.

Importantly, DGE appears to be associated with other major complications, including POPF [40]. Accordingly, the present analysis found lower rates of both overall and clinically relevant POPF in the BEE group. However, this pattern was less consistent than for DGE, as biochemical leak and the individual Grade B and Grade C estimates did not differ, so a comparable severity-shifting effect on POPF cannot be asserted from the present data. This extends beyond an existing meta-analysis in which POPF did not differ between groups, likely reflecting the smaller number of studies available at that time [13]. Any mechanistic explanation for the observed reduction in POPF remains speculative, as none of the included studies directly assessed these proposed pathways. It has been suggested that BEE allows the diversion of biliopancreatic secretions, reducing intraluminal pressure within the afferent limb [14, 23]. This has further been hypothesized to protect the pancreatic remnant from the corrosive and inflammatory effects of pancreatic enzymes mixed with bile and intestinal contents [28]. Furthermore, several factors, such as anastomotic edema, gastric acid irritation, limb volvulus, or adhesions, can contribute to anastomotic obstruction. This could theoretically increase the risk of proximal fistula formation, due to the increased biliopancreatic outflow pressure [14, 23]. BEE may enhance the stability of the afferent and efferent limbs of the gastrojejunostomy, reducing the tendency for torsion and angulation, further reducing the chance of obstruction and promoting gastric emptying [27]. Given that the reduction in POPF was confined to the composite overall and clinically relevant estimates, with no differences in biochemical leak or individual POPF grades, and that the overall POPF estimate was sensitive to single-study exclusion, these mechanistic considerations should be regarded as hypothesis-generating.

Our analysis found that the operative time was longer in patients undergoing PD with BEE. This is an expected and quantifiable outcome of adding an additional anastomosis, although it likely remains clinically acceptable in relation to the total duration of PD and no difference in serious morbidity. The shorter LOS observed in the BEE group is likely a clinical manifestation of reducing Grade B and C DGE and their sequelae. In keeping with this, Hochwald et al. documented earlier resumption of oral feeding and a shorter median LOS with BEE [11]. Notably, Carmel et al. demonstrated a shorter LOS in the BEE group even within a structured ERAS protocol, suggesting that BEE provides benefit independent of and additive to pathway-based perioperative optimization [10]. The high statistical heterogeneity in the LOS analysis most likely reflects institutional variation in discharge criteria, ERAS adoption, and DGE management protocols. Collectively, these findings suggest that BEE confers additional benefits even within established ERAS pathways; as such, it may warrant consideration for integration into contemporary practice for selected patients. While further studies are required to define patient selection criteria, BEE should be considered in patients with increased risk of DGE, including PPPD cases and cases with high-risk pancreatic remnants [31, 41, 42]. Despite these findings, BEE has not been widely adopted after PD. This likely reflects the absence of convincing randomized evidence, the lack of a standardized technique, and the availability of competing strategies for DGE prevention such as antecolic reconstruction, pylorus preservation, and ERAS pathways. Therefore, the use of BEE is commonly determined by surgeon preference rather than protocol. The barrier to adoption thus appears to lie less in concern about harm than in the absence of standardization and high-certainty evidence of benefit. A further consideration is that the available evidence derives predominantly from open PD, reflecting the era of the included studies rather than a restriction of the technique. As BEE is a reconstructive maneuver performed after the resection and is independent of the access route, the trade-off it presents remains relevant to laparoscopic and robotic practice, in which DGE and POPF likewise dominate postoperative morbidity. This research question therefore remains of importance as minimally invasive surgeons continue to weigh the addition of a Braun anastomosis at the time of reconstruction, although dedicated evaluation within laparoscopic and robotic cohorts is warranted.

This study has several limitations that merit consideration. First, most included studies were retrospective and conducted in single-institution settings, introducing potential selection bias and limiting generalizability. Second, high heterogeneity was observed in operative time, LOS, and several DGE grade analyses, likely reflecting variation in surgical technique, reconstruction route, BEE distance from the gastrojejunostomy, loop length, surgeon experience, and perioperative protocols across included studies. In addition, the reconstruction technique was inconsistently reported across the included studies and was insufficiently stratified by treatment arm, precluding subgroup analysis. As the method of pancreatic and gastric reconstruction is itself a determinant of DGE and POPF, residual confounding of the pooled estimates cannot be excluded. Third, the proportion of malignant indications was lower in patients with BEE. This imbalance was largely attributable to a single large study [24], with balanced proportions between the arms after its exclusion and unchanged pooled estimates in the leave-one-out analysis. Nonetheless, pooling of unadjusted estimates remains susceptible to residual confounding. Fourth, the BEE distance from the gastrojejunostomy varied from 10 to 45 cm across studies, and this technical heterogeneity may explain some of the inconsistency in DGE outcomes, as distance and limb configuration plausibly influence the degree of afferent limb decompression. Additionally, the ISGPS definitions for both DGE and POPF have evolved over time, though the core diagnostic criteria have remained largely consistent. Differences in the definitions of bile leak and pancreas texture were not widely reported or standardized among included studies, potentially introducing classification bias. Kakaei et al. [22] applied a non-ISGPS definition of DGE, introducing further outcome-definition heterogeneity. Moreover, Xu et al. [14] and Meng et al. [28] originate from the same institution with overlapping study periods. Although the reported enrollment windows and mutually exclusive pancreatojejunostomy techniques indicate largely distinct populations, minimal patient-level overlap cannot be definitively excluded. Finally, the long-term outcomes of patients were not reported across studies, precluding formal analysis. Despite these limitations, this analysis provides the most comprehensive synthesis of available evidence to date and incorporates four RCTs, strengthening the reliability of the primary findings.

## Conclusion

This updated meta-analysis demonstrates that BEE appears to be a safe and potentially beneficial adjunct to PD for selected patients. The addition of BEE appears to be associated with a reduction in DGE and POPF without a meaningful difference in overall postoperative morbidity or mortality. These findings were not confirmed in a subgroup analysis of four RCTs and should be interpreted with caution. Further adequately powered RCTs are warranted to confirm these findings and to better define the selection criteria for patients most likely to benefit from the addition of BEE to PD.

## Supplementary Information

Below is the link to the electronic supplementary material.

Supplementary file1 (DOCX 16 KB)

Supplementary file2 (DOCX 40 KB)

Supplementary file3 (DOCX 1217 KB)

Supplementary file4 (DOCX 21 KB)

Supplementary file5 (DOCX 654 KB)

## Data Availability

The dataset used for this meta-analysis will be shared upon request from the study authors.

## Abbreviations

ASA: American society of anesthesiologists
BEE: Braun enteroenterostomy
BMI: Body mass index
DGE: Delayed gastric emptying
ERAS: Enhanced recovery after surgery
EBL: Estimated blood loss
GJ: Gastrojejunostomy
GRADE: Grading of recommendations, assessment, development, and evaluation
ICU: Intensive care unit
ISGPS: International study group for pancreatic surgery
LOS: Length of stay
NGT: Nasogastric tube
PD: Pancreatoduodenectomy
POPF: Postoperative pancreatic fistula
PPPD: Pylorus-preserving pancreatoduodenectomy
PRISMA: Preferred reporting items for systematic reviews and meta-analyses
RCT: Randomized controlled trial
ROB-2: Risk of bias tool for randomized trials
ROBINS-I: Risk of bias in non-randomized studies of interventions
SSPPD: Subtotal stomach-preserving pancreatoduodenectomy
