# Supplementary material for: Efficacy and safety of Braun enteroenterostomy in patients undergoing pancreatoduodenectomy: a systematic review and meta-analysis
Source: Surg Endosc. 2026 Sep 23;40(10):8383–99. doi: 10.1007/s00464-026-13370-8 (PMC13633350; doi:10.1007/s00464-026-13370-8)
Supplement: Supplementary file 1 [file 464_2026_13370_MOESM1_ESM.docx]

**Supplementary Item 1:** Database Search Strategy used.

**Pubmed**

"pancreaticoduodenectomy"[MeSH Terms] OR "pancreaticoduodenectomy"[MeSH Terms]) OR ("duodenopancreatectomy" OR "pancreaticoduodenectomy" OR "pancreatoduodenal resection" OR "pancreatoduodenectomy" OR "pancreatoduodenal resection" OR "pancreatoduodenectomy" OR "total pancreatic duodenectomy" OR "Whipple operation" OR "Whipple procedure" OR "Whipple resection" OR "pancreaticoduodenectomy" AND "Braun enteroenterostomy" OR Braun OR enteroenterostomy OR jejunojejunostomy

**Embase**

#7. #3 AND #6

#6. #4 OR #5

#5. 'braun enteroenterostomy' OR braun OR enteroenterostomy OR jejunojejunostomy

#4. 'jejunojejunostomy'/exp

#3. #1 OR #2

#2. 'duodenopancreatectomy' OR 'pancreaticoduodenectomy' OR 'pancreatoduodenal resection' OR 'pancreatoduodenectomy' OR 'pancreatoduodenal resection' OR 'pancreatoduodenectomy' OR 'total pancreatic duodenectomy' OR 'whipple operation' OR 'whipple procedure' OR 'whipple resection' OR 'pancreaticoduodenectomy'

#1. 'pancreaticoduodenectomy'/exp

**Cochrane**

#1: MeSH descriptor: [Pancreaticoduodenectomy] explode all trees

#2: ("duodenopancreatectomy" OR "pancreaticoduodenectomy" OR "pancreatoduodenal resection" OR "pancreatoduodenectomy" OR "pancreatoduodenal resection" OR "pancreatoduodenectomy" OR "total pancreatic duodenectomy" OR "Whipple operation" OR "Whipple procedure" OR "Whipple resection" OR "pancreaticoduodenectomy"):ti,ab,kw (Word variations have been searched)

#3: #1 OR #2

#4: ("Braun enteroenterostomy" OR Braun OR enteroenterostomy OR jejunojejunostomy):ti,ab,kw (Word variations have been searched)

#5: #3 AND #4

**CiNAHL**

(MM "Pancreaticoduodenectomy" OR ("duodenopancreatectomy" OR "pancreaticoduodenectomy" OR "pancreatoduodenal resection" OR "pancreatoduodenectomy" OR "pancreatoduodenal resection" OR "pancreatoduodenectomy" OR "total pancreatic duodenectomy" OR "Whipple operation" OR "Whipple procedure" OR "Whipple resection" OR "pancreaticoduodenectomy")) AND ("Braun enteroenterostomy" OR Braun OR enteroenterostomy OR jejunojejunostomy)

**Scopus**

( TITLE-ABS-KEY ( "duodenopancreatectomy" OR "pancreaticoduodenectomy" OR "pancreatoduodenal resection" OR "pancreatoduodenectomy" OR "pancreatoduodenal resection" OR "pancreatoduodenectomy" OR "total pancreatic duodenectomy" OR "Whipple operation" OR "Whipple procedure" OR "Whipple resection" OR "pancreaticoduodenectomy" ) ) AND ( TITLE-ABS-KEY ( "Braun enteroenterostomy" OR Braun OR enteroenterostomy OR jejunojejunostomy ) )

**Web of Science Core Collection**

1: TS=("duodenopancreatectomy" OR "pancreaticoduodenectomy" OR "pancreatoduodenal resection" OR "pancreatoduodenectomy" OR "pancreatoduodenal resection" OR "pancreatoduodenectomy" OR "total pancreatic duodenectomy" OR "Whipple operation" OR "Whipple procedure" OR "Whipple resection" OR "pancreaticoduodenectomy")

2: TS=("Braun enteroenterostomy" OR Braun OR enteroenterostomy OR jejunojejunostomy)

3: #1 AND #2
