# Supplementary material for: Efficacy and safety of Braun enteroenterostomy in patients undergoing pancreatoduodenectomy: a systematic review and meta-analysis
Source: Surg Endosc. 2026 Sep 23;40(10):8383–99. doi: 10.1007/s00464-026-13370-8 (PMC13633350; doi:10.1007/s00464-026-13370-8)
Supplement: Supplementary file 2 [file 464_2026_13370_MOESM2_ESM.docx]

**Supplementary Item 2:** Preferred Reporting Items for Systematic Reviews (PRISMA) Flowchart.

**Identification of studies via databases and registers**

Records removed *before screening*:

Duplicate records removed (n = 57)

Records identified from:

Databases (n = 340)

Registers (n = 0)

**Identification**

Records screened

(n = 283)

Records excluded

(n = 218)

Reports sought for retrieval

(n = 65)

Reports not retrieved

(n = 0)

**Screening**

Reports assessed for eligibility

(n = 65)

Reports excluded:

Abstract only (n = 23)

Wrong intervention (n = 8)

Wrong comparator (n = 6)

Wrong language (n = 4)

Wrong outcomes (n=2)

Wrong patient population (n=2)

Wrong study design (n=2)

Duplicate data set (n=1)

Studies included in review

(n = 17)

Reports of included studies

(n = 17)

**Included**
