# Supplementary material for: Efficacy and safety of Braun enteroenterostomy in patients undergoing pancreatoduodenectomy: a systematic review and meta-analysis
Source: Surg Endosc. 2026 Sep 23;40(10):8383–99. doi: 10.1007/s00464-026-13370-8 (PMC13633350; doi:10.1007/s00464-026-13370-8)
Supplement: Supplementary file 3 [file 464_2026_13370_MOESM3_ESM.docx]

**Supplementary Item 3:** A: Risk of bias in non-randomized studies of interventions (ROBINS-I) assessment, B: Risk of bias for randomized trials (RoB 2).

A.

B.
