# Supplementary material for: Efficacy and safety of Braun enteroenterostomy in patients undergoing pancreatoduodenectomy: a systematic review and meta-analysis
Source: Surg Endosc. 2026 Sep 23;40(10):8383–99. doi: 10.1007/s00464-026-13370-8 (PMC13633350; doi:10.1007/s00464-026-13370-8)
Supplement: Supplementary file 4 [file 464_2026_13370_MOESM4_ESM.docx]

**Supplementary Item 4:** GRADE evidence table for postoperative outcomes following pancreatoduodenectomy with and without Braun Enteroenterostomy.

| **Certainty assessment** | | | | | | | **№ of patients** | | **Effect** | | **Certainty** | **Importance** |
| --- | --- | --- | --- | --- | --- | --- | --- | --- | --- | --- | --- | --- |
| **№ of studies** | **Study design** | **Risk of bias** | **Inconsistency** | **Indirectness** | **Imprecision** | **Other considerations** | **BEE** | **Controls** | **Relative (95% CI)** | **Absolute (95% CI)** |  |  |
|  | | | | | | | | | | | | |
| **DGE Overall** | | | | | | | | | | | | |
| 15 | randomised trials and observational studies | serious | not serious | not serious | not serious | none | 154/1266 (12.2%) | 283/1043 (27.1%) | **OR 0.39** (0.28 to 0.54) | **145 fewer per 1,000** (from 177 fewer to 104 fewer) | ⨁⨁◯◯ Low | CRITICAL |
| **DGE B+C** | | | | | | | | | | | | |
| 13 | randomised trials and observational studies | serious | not serious | not serious | not serious | none | 96/1227 (7.8%) | 190/1008 (18.8%) | **OR 0.36** (0.23 to 0.57) | **111 fewer per 1,000** (from 138 fewer to 72 fewer) | ⨁⨁◯◯ Low | CRITICAL |
| **POPF Overall** | | | | | | | | | | | | |
| 15 | randomised trials and observational studies | serious | not serious | not serious | not serious | none | 193/1254 (15.4%) | 271/1072 (25.3%) | **OR 0.68** (0.49 to 0.94) | **66 fewer per 1,000** (from 111 fewer to 12 fewer) | ⨁⨁◯◯ Low | IMPORTANT |
| **POPF B+C** | | | | | | | | | | | | |
| 13 | randomised trials and observational studies | serious | not serious | not serious | not serious | none | 94/864 (10.9%) | 169/957 (17.7%) | **OR 0.60** (0.41 to 0.88) | **63 fewer per 1,000** (from 96 fewer to 18 fewer) | ⨁⨁◯◯ Low | IMPORTANT |
| **Clavien-Dindo ≥ III** | | | | | | | | | | | | |
| 5 | randomised trials and observational studies | serious | not serious | not serious | Serious^c^ | none | 45/293 (15.4%) | 91/356 (25.6%) | **OR 0.66** (0.36 to 1.19) | **71 fewer per 1,000** (from 146 fewer to 34 more) | ⨁⨁◯◯ Low | CRITICAL |
| **Operative time** | | | | | | | | | | | | |
| 14 | randomised trials and observational studies | serious | Serious^b^ | not serious | not serious | none | 966 | 1169 | - | MD **12.57 higher** (0.88 higher to 24.26 higher) | ⨁⨁◯◯ Low | IMPORTANT |
| **Hospital stay** | | | | | | | | | | | | |
| 11 | randomised trials and observational studies | serious | Serious^b^ | not serious | not serious | none | 766 | 877 | - | MD **3.36 lower** (5.59 lower to 1.13 lower) | ⨁⨁◯◯ Low | IMPORTANT |

**BEE:** Braun Enteroenterostomy**, CI:** confidence interval; **MD:** mean difference; **OR:** odds ratio

#### Explanations

a. The majority of included studies were rated at a serious risk of bias.

b. The presence of heterogeneity throughout the main analysis may compromise the consistency of the results.

c. The confidence interval crosses the line of no effect and spans from no difference in risk to a moderate decrease
