# Supplementary material for: Efficacy and safety of Braun enteroenterostomy in patients undergoing pancreatoduodenectomy: a systematic review and meta-analysis
Source: Surg Endosc. 2026 Sep 23;40(10):8383–99. doi: 10.1007/s00464-026-13370-8 (PMC13633350; doi:10.1007/s00464-026-13370-8)
Supplement: Supplementary file 5 [file 464_2026_13370_MOESM5_ESM.docx]

**Supplementary Item 5.** Leave-one-out sensitivity analysis for overall DGE, clinically relevant DGE, overall POPF, and clinically relevant POPF. Values are pooled odds ratios (95% confidence intervals); I², recalculated after exclusion of the indicated study (Mantel–Haenszel random-effects model).

| **Study omitted** | **Overall DGE** | **Clinically relevant DGE** | **Overall POPF** | **Clinically relevant POPF** |
| --- | --- | --- | --- | --- |
| **None (primary analysis)** | 0.39 (0.28–0.54); 33% | 0.36 (0.23–0.57); 45% | 0.68 (0.49–0.94); 42% | 0.60 (0.41–0.88); 34% |
| **Carmel et al.** | 0.37 (0.26–0.52); 33% | 0.34 (0.21–0.56); 45% | 0.65 (0.46–0.93); 42% | 0.59 (0.38–0.90); 38% |
| **Cordesmeyer et al.** | 0.40 (0.28–0.56); 37% | 0.33 (0.22–0.51); 36% | 0.69 (0.50–0.97); 45% | 0.62 (0.41–0.92); 39% |
| **Fujieda et al.** | 0.38 (0.27–0.53); 36% | 0.35 (0.21–0.56); 47% | 0.68 (0.48–0.97); 46% | 0.60 (0.40–0.91); 39% |
| **Hochwald et al.** | 0.39 (0.27–0.56); 38% | 0.39 (0.24–0.63); 45% | NA | 0.57 (0.38–0.84); 33% |
| **Hwang et al.** | 0.39 (0.27–0.55); 38% | 0.38 (0.24–0.60); 47% | 0.69 (0.49–0.97); 46% | 0.59 (0.40–0.86); 36% |
| **Kakaei et al.** | 0.38 (0.27–0.54); 38% | NA | 0.69 (0.49–0.96); 45% | NA |
| **Luu et al.** | NA | NA | 0.66 (0.47–0.94); 44% | 0.57 (0.38–0.85); 34% |
| **Marimuthu et al.** | 0.37 (0.26–0.53); 36% | 0.37 (0.23–0.60); 49% | 0.70 (0.49–0.99); 45% | 0.62 (0.41–0.93); 39% |
| **Meng et al.** | 0.41 (0.30–0.56); 27% | 0.39 (0.25–0.60); 42% | **0.74 (0.54–1.01); 32%*** | 0.66 (0.45–0.96); 26% |
| **Nikfarjam et al.** | 0.40 (0.29–0.55); 31% | NA | 0.68 (0.49–0.97); 46% | 0.62 (0.41–0.92); 39% |
| **Tanaka et al.** | 0.39 (0.28–0.55); 38% | 0.36 (0.23–0.58); 49% | 0.66 (0.46–0.94); 44% | 0.60 (0.40–0.91); 40% |
| **Usenko et al.** | 0.39 (0.27–0.55); 38% | 0.36 (0.22–0.58); 49% | 0.65 (0.46–0.92); 43% | 0.56 (0.38–0.82); 30% |
| **Vutukuru et al.** | 0.36 (0.26–0.49); 20% | 0.34 (0.21–0.53); 42% | 0.66 (0.46–0.94); 44% | 0.58 (0.39–0.86); 36% |
| **Watanabe et al.** | 0.41 (0.30–0.57); 30% | 0.39 (0.25–0.63); 44% | 0.67 (0.47–0.96); 45% | NA |
| **Xu et al.** | 0.44 (0.32–0.60); 16% | 0.40 (0.25–0.64); 37% | **0.78 (0.60–1.01); 7%*** | 0.70 (0.50–0.96); 0% |
| **Zhang et al.** | 0.37 (0.26–0.53); 35% | 0.34 (0.21–0.55); 45% | 0.64 (0.47–0.87); 33% | NA |
| **Xu et al. + Zhang et al.** | 0.42 (0.30–0.59); 19% | 0.38 (0.22–0.64); 40% | 0.75 (0.58–0.96); 0% | 0.70 (0.50–0.96); 0%† |

*: statistical significance lost.

†: Zhang et al. was not included in the clinically relevant POPF analysis; this estimate reflects exclusion of Xu et al. only.

NA: Not applicable. CR: Clinically-relevant, DGE: Delayed gastric emptying, POPF: Postoperative pancreatic fistula.
